# AlphaBet: Combination of Radium-223 and [^17^^7^Lu]Lu-PSMA-I&T in men with metastatic castration-resistant prostate cancer (clinical trial protocol)

**DOI:** 10.3389/fmed.2022.1059122

**Published:** 2022-11-18

**Authors:** Louise Kostos, James P. Buteau, Theresa Yeung, Juliana Di Iulio, Jing Xie, Anthony Cardin, Kwang Y. Chin, Brittany Emmerson, Katie L. Owen, Belinda S. Parker, Heidi Fettke, Luc Furic, Arun A. Azad, Michael S. Hofman

**Affiliations:** ^1^Department of Medical Oncology, Peter MacCallum Cancer Centre, Melbourne, VIC, Australia; ^2^Sir Peter MacCallum Department of Oncology, University of Melbourne, Melbourne, VIC, Australia; ^3^Molecular Imaging and Therapeutic Nuclear Medicine, Peter MacCallum Cancer Centre, Melbourne, VIC, Australia; ^4^Prostate Cancer Theranostics and Imaging Centre of Excellence, Peter MacCallum Cancer Centre, Melbourne, VIC, Australia; ^5^Centre for Biostatistics and Clinical Trials (BaCT), Peter MacCallum Cancer Centre, Melbourne, VIC, Australia; ^6^Department of Cancer Imaging, Peter MacCallum Cancer Centre, Melbourne, VIC, Australia; ^7^Cancer Evolution and Metastasis Program, Peter MacCallum Cancer Centre, Melbourne, VIC, Australia; ^8^Cancer Research Division, Peter MacCallum Cancer Centre, Melbourne, VIC, Australia

**Keywords:** metastatic castration-resistant prostate cancer, alpha therapy, micrometastatic disease, ^177^Lu-PSMA, radium-223, PSMA

## Abstract

**Background:**

[^177^Lu]Lu-PSMA is a radioligand therapy used in metastatic castration-resistant prostate cancer (mCRPC). Despite a survival benefit, the responses for many patients receiving [^177^Lu]Lu-PSMA are not durable, and all patients eventually develop progressive disease. The bone marrow is the most common site of progression. Micrometastases in this area likely receive an inadequate dose of radiation, as the emitted beta-particles from ^177^Lu travel an average range of 0.7 mm in soft tissue, well beyond the diameter of micrometastases. Radium-223 (^223^Ra) is a calcium-mimetic and alpha-emitting radionuclide approved for use in men with mCRPC with bone metastases. The range of emitted alpha particles in soft tissue is much shorter (≤100 μm) with high linear energy transfer, likely more lethal for osseous micrometastases. We anticipate that combining a bone-specific alpha-emitter with [^177^Lu]Lu-PSMA will improve eradication of micrometastatic osseous disease, and thereby lead to higher and longer responses.

**Methods:**

This is a single-center, single-arm phase I/II trial evaluating the combination of ^223^Ra and [^177^Lu]Lu-PSMA-I&T in men with mCRPC. Thirty-six patients will receive 7.4 GBq of [^177^Lu]Lu-PSMA-I&T, concurrently with ^223^Ra in escalating doses (28 kBq/kg – 55kBq/kg), both given intravenously every six weeks for up to six cycles. Eligible patients will have at least two untreated bone metastases visible on bone scintigraphy, and PSMA-positive disease on PSMA PET scan. Patients must have adequate bone marrow and organ function and be willing to undergo tumor biopsies. Patients with discordant disease visible on FDG PET scan (defined as FDG positive disease with minimal or no PSMA expression and no uptake on bone scan) will be excluded. Other key exclusion criteria include the presence of diffuse marrow disease, prior treatment with ^223^Ra or [^177^Lu]Lu-PSMA, or more than one prior line of chemotherapy for prostate cancer. The co-primary objectives of this study are to determine the maximum tolerated dose of ^223^Ra when combined with [^177^Lu]Lu-PSMA-I&T and the 50% PSA response rate.

**Conclusion:**

The AlphaBet trial is a phase I/II study combining ^223^Ra with [^177^Lu]Lu-PSMA-I&T in patients with mCRPC. We aim to enroll the first patient in Q3 2022, and recruitment is anticipated to continue for 24 months.

**Study registration:**

NCT05383079.

## Background

One of the recent practice changes for mCRPC, a leading cause of cancer-related death worldwide (1), has been the integration of [^177^Lu]Lu-PSMA into the post-taxane and androgen receptor inhibitor (ARI) treatment paradigm. [^177^Lu]Lu-PSMA is a form of radionuclide therapy whereby the isotope lutetium-177 (^177^Lu) is attached to a prostate-specific membrane antigen (PSMA) radioligand to enable targeted delivery of radiation to prostate cancer cells *via* beta-particle emission. The landmark TheraP trial compared the use of [^177^Lu]Lu-PSMA-617 with cabazitaxel in patients with mCRPC and found greater PSA responses (66 vs. 37% by intention to treat), a reduction in pain scores, and fewer grade 3 or higher adverse events (AEs) in the [^177^Lu]Lu-PSMA-617 arm (2). [^177^Lu]Lu-PSMA-617 was proven to extend overall survival (OS) as well as progression-free survival (PFS) in the VISION trial, where it was compared to protocol-defined best standard care alone (3). Both the TheraP and VISION trials utilized [^68^Ga]Ga-PSMA-11 PET/CT for patient selection, with TheraP requiring a higher intensity of uptake of SUVmax greater than or equal to 20, compared to greater than liver in VISION. TheraP additionally used 2-[18F]fluoro-2-deoxy-D-glucose (FDG) PET/CT to identify sites of PSMA-negative disease whereas VISION used contrast-enhanced CT alone. Following publication of the VISION results, [^177^Lu]Lu-PSMA-617 has been approved by the Food and Drug Administration (FDA) for use in the post-taxane, post-ARI mCRPC setting.

Several forms of PSMA-directed therapy exist in addition to PSMA-617, including the radioligand PSMA-I&T and monoclonal antibody J591. Comparing PSMA-I&T and PSMA-617, they are almost identical peptides with the main difference being the chemical chelator that binds the radioactive element and PSMA receptor binding structure. Dosimetry data demonstrates comparable absorbed doses and retrospective analyses suggest similar toxicities and clinical responses (4, 5). The European Association of Nuclear Medicine (EANM) radionuclide therapy guidelines apply to both [^177^Lu]Lu-PSMA-617 and [^177^Lu]Lu-PSMA-I&T (5).

Long-term follow-up of the 50 patients enrolled in the LuPSMA trial (6), the first phase II trial evaluating [^177^Lu]Lu-PSMA-617 in men with mCRPC, found that all patients eventually developed PSA progression, even if they had an initial complete or exceptional response on post-therapy SPECT/CT. The majority of patients (56%) developed progressive bone marrow disease (7). The inability to deliver lethal doses of radiation to micrometastatic sites such as in the bone marrow may be a contributing reason for the lack of durable response for many patients. ^177^Lu releases relatively low linear energy transfer (LET) (0.2 keV/μm) beta radiation, which usually results in single-stranded DNA (ssDNA) breaks. Single metastatic cells or small cell clusters may not receive adequate radiation to result in cell death, owing to the lack of cross-fire effect which normally occurs in macro-tumors where there are abundant neighboring cells.

Alternative radionuclides with a higher LET may overcome this by inducing cytotoxic double-stranded DNA (dsDNA) breaks, leading to more robust treatment of micrometastatic disease. Alpha-emitters are one such example, which generally have a short path-length and high LET compared to beta-emitters, making them ideal for treating micrometastases. Usually only a few alpha particles through a cell nucleus are sufficient to induce cell death, and due to the short path length, bystander radiation is minimal. Examples of clinically available alpha-emitters include bismuth-213 (^213^Bi), astatine-221 (^221^At) and lead-212 (^212^Pb). Limitations of these alpha-emitters, however, are the short half-life (t_1/2_, 7.2 h for ^221^At, 10.6 hours for ^212^Pb, and 45.6 min for ^213^Bi), making treatment of cancer cells in solid tumors where deep penetration is required or less accessible sites a challenge. To overcome this, several other alpha-emitters were introduced to the clinic with longer half-lives, including radium-223 (^223^Ra, t_1/2_ = 11.4 days) and actinium-225 (^225^Ac, t_1/2_ = 10.0 days) (8). There are several studies ongoing evaluating the combination of alpha-emitters with a PSMA-based radioligand (NCT04597411, NCT05219500) or monoclonal antibody (NCT04886986). Preliminary data from an ^225^Ac radionuclide compounded with J591 looks promising in terms of safety and efficacy (9). Unfortunately, several factors limit mass distribution of some targeted alpha therapies including complex radiochemistry and production leading to limited supply.

^223^Ra is a calcium-mimetic alpha-emitter, with targeted activity against bone metastases. It has been studied extensively in mCRPC and is FDA approved for use in patients with bone-metastases and no visceral disease. Consequently, it is readily available and delivered in a pre-formulated vial (unlike other alpha-emitters). The short path length of <100 μm and high LET of 80 KeV/μm make ^223^Ra ideal for treating osseous micrometastases. In a phase II dose-finding study of ^223^Ra, patients received one of three differing doses of ^223^Ra−25 kBq/kg, 50 kBq/kg, and 80 kBq/kg. There was no difference in hematological toxicity amongst the three cohorts, with a low frequency of grade 2 or higher adverse events overall. The dose of 50 kBq/kg was selected for future studies. In the practice-changing phase III ALSYMPCA trial, ^223^Ra was delivered at a dose of 50 kBq/kg intravenously every 4 weeks for up to 6 doses (10). Compared to placebo, treatment with ^223^Ra was associated with an improvement in median OS (14.9 months vs. 11.3 months, HR 0.70) (10). ^223^Ra was well tolerated with fewer AEs compared to placebo and improved quality of life (QoL) scores. For ^223^Ra, the incidence of grade 3 or higher anemia, neutropaenia and thrombocytopaenia was 13, 3 and 6%, respectively (vs. 13, 3 and 1% in the placebo arm). Pathologic fractures occurred in 4% of patients receiving ^223^Ra compared to 5% in the placebo arm.

A reassessment of the primary standardization of ^223^Ra radioactivity measurement was initiated by the US National Institute of Standards and Technology (NIST) in 2015 (11). A discrepancy of approximately 10% between the initial published NIST primary standardization (12) and this assessment was identified, and as a result the recommended dose of ^223^Ra was adjusted from 50 kBq/kg to 55 kBq/kg every 4 weeks (11).

^223^Ra has been studied in combination with a variety of other anti-cancer therapies including chemotherapy, anti-androgen therapy, immunotherapy, and PARP inhibitors for the treatment of advanced prostate cancer (see Table 1). In the pivotal studies evaluating ^223^Ra in combination with second-generation anti-androgens, a significantly increased fracture risk was an unexpected finding. In the phase III ERA-223 trial, patients with mCRPC received abiraterone acetate plus prednisolone in combination with ^223^Ra, vs. abiraterone acetate alone (13). OS did not differ significantly between groups, but the combination arm was associated with increased fracture risk (28.6 vs. 11.4%) leading to premature unblinding of the trial. The EORTC 1333/PEACEIII trial evaluates the addition of ^223^Ra to enzalutamide in mCRPC patients (14). On safety analysis, it was noted that, similarly to the ERA-223 trial, the fracture risk was significantly increased in the group who received enzalutamide in combination with ^223^Ra, without concomitant bone protective treatment. Following the results of the ERA-223 study, however, the EORTC 1333 study was amended, and bisphosphonate treatment was then mandated for all patients. Following this, the fracture rate significantly decreased in both arms of the study. Recruitment continues and efficacy outcomes are awaited.

**Table 1:** Combination studies with ^223^Ra in patients with mCRPC.

**Clinical trial registration number**	**Intervention**	**Phase**	**Primary outcome**	**Results**
**Chemotherapy**				
NCT03737370	Radium-223 55 kBq/kg Q4W + Docetaxel Q2W (escalating doses)	I	Incidence of DLTs	NA, recruitment ongoing
NCT03574571	Radium-223 55 kBq/kg Q6W + Docetaxel 60 mg/m^2^ Q3W X 10 vs. Docetaxel 75 mg/m^2^ Q3W alone	III	OS	NA, recruitment ongoing
NCT01106352	Radium-223 50 kBq/kg Q6W + Docetaxel 60 mg/m^2^ Q3W X 10 vs. Docetaxel 75 mg/m^2^ Q3W alone	I/II	Incidence of DLTs and AEs	The RP2D for the combination was radium-223 55 kBq/kg Q6W × 5 doses, + docetaxel 60 mg/m^2^ Q3W × 10 doses. Median time to PSA progression favored the combination (6.6 vs. 4.8 m)
**Immunotherapy**				
NCT03093428	Radium-223 55 kBq/kg Q4W + Pembrolizumab 200 mg Q3W vs. Radium-223 55 kBq/kg Q4W alone	II	Number of Participants with Increased Immune Cell Infiltration Across Arms	No difference between Arm A and B in rPFS (6.7 m vs. 5.7 m) or OS (16.9 vs. 16.0 m) No evidence of increased CD4+ or CD8+ T-cell infiltration in Arm A
NCT04109729	Radium-223 55 kBq/kg Q4W + Nivolumab 480 mg Q4W	I/II	Safety, ctDNA reduction after 6 weeks of nivolumab treatment	NA, recruitment ongoing
NCT02814669	Radium-223 55 kBq/kg Q4W + Atezolizumab 480 mg Q2W	I	Incidence of DLTs and AEs, ORR	ORR 6.3%, Median rPFS 3.0 m, Median OS 16.3 m No clear evidence of benefit with increased toxicity in combination than either drug alone
NCT04071236	Radium-223 Q4W x 6 + Peposertib +/- Avelumab Q2W vs. Radium-223 Q4W x 6 alone	I/II	Incidence of DLTs, rPFS	NA, recruitment ongoing
NCT02463799	Radium-223 50 kBq/kg Q4W + Sipuleucel-T Q2W vs. Sipuleucel-T Q2W alone	II	Immune responses to treatment with Sipuleucel-T measured by peripheral PA2024 T-cell proliferation	Higher 50% PSA response rate (31 vs. 0%) and longer PFS (39 vs. 12 w) and OS (NR vs. 2.6 y) seen in combination arm
**Anti-androgen therapy**				
NCT02199197	Radium-223 55 kBq/kg Q4W + Enzalutamide 160 mg daily vs. Enzalutamide alone	II	Incidence of AEs, change in serum N-telopeptides from baseline	No statistically significant difference in OS, rPFS, PSA PFS PSA PFS2 improved with combination (18.7 vs. 8.4 m)
NCT02194842	Radium-223 55 kBq/kg Q4W + Enzalutamide 160 mg daily vs. Enzalutamide alone	III	rPFS	NA, recruitment ongoing
NCT02043678 (ERA-223)	Radium-223 55 kBq/kg Q4W + Abiraterone Acetate 1000 mg daily and Prednisolone vs. Abiraterone alone	III	Symptomatic skeletal event free survival	No improvement in OS or median symptomatic skeletal event-free survival
**PARP inhibitors**				
NCT03317392	Radium-223 Q4W + Olaparib	I/II	MTD of Radium-223 and Olaparib, rPFS	NA, recruitment ongoing
NCT03076203	Radium-223 Q4W + Niraparib	I	MTD	The MTD of Niraparib was 100 mg in the chemo-exposed arm and 200 mg in the chemo-naïve arm

Similarly, ^223^Ra has been combined with docetaxel chemotherapy (15). In a phase I trial, 20 patients were enrolled and received up to 5 doses of ^223^Ra given every 6 weeks, and docetaxel every 3 weeks. The starting dose of ^223^Ra was 27.5 kBq/kg and was then escalated to 55 kBq/kg if tolerated. Docetaxel was given at a dose of 75 mg/m^2^ which is the standard therapeutic dose, with a plan to reduce to 60 mg/m^2^ in the event of a dose-limiting toxicity (DLT). Febrile neutropaenia was dose limiting and therefore the recommended phase II dose (RP2D) for the combination was ^223^Ra 55 kBq/kg every 6 weeks × 5 doses, plus docetaxel 60 mg/m^2^ every 3 weeks × 10 doses. In the phase II study, which compared this combination to docetaxel alone, the combination arm had more durable suppression of PSA (median time to PSA progression, 6.6 vs. 4.8 months, respectively) and alkaline phosphatase (ALP) (median time to ALP progression 9 vs. 7 months).

Though ^223^Ra has not previously been combined with [^177^Lu]Lu-PSMA, sequential alpha/beta-emitting therapy using ^177^Lu and ^223^Ra has previously been studied in both prospective and retrospective analyses (16–18). Sartor et al. analyzed safety data from patients who were administered [^177^Lu]Lu-PSMA following treatment with ^223^Ra (19). Twenty-six patients from a real-world patient registry (REASSURE study) were included in this analysis. The median time between the two treatments was 8 months (range 1–31). Five patients had Grade 3 or higher haematologic AEs during or after treatment with [^17^^7^Lu]Lu-PSMA, most commonly anemia. Overall, though this was a small patient sample, there were no apparent new safety signals.

Similarly, Baumgarten et al. explored the safety of [^177^Lu]Lu-PSMA when given immediately after ^223^Ra in a retrospective analysis. Twenty-nine patients were studied who received [^177^Lu]Lu-PSMA within 5 weeks (±3 weeks) of ^223^Ra injection. Grade 3-4 anemia necessitating a blood transfusion was seen in 5 patients, 2 patients required a dose-reduction and 7 patients discontinued treatment due to significant cytopaenias. Following this analysis, the authors concluded that treatment with [^17^^7^Lu]Lu-PSMA within 12 weeks of ^223^Ra had an acceptable risk profile (20). The retrospective WARMTH and RALU studies corroborated prior data and found that sequential therapy was feasible and well-tolerated (21).

**Figure 1:**
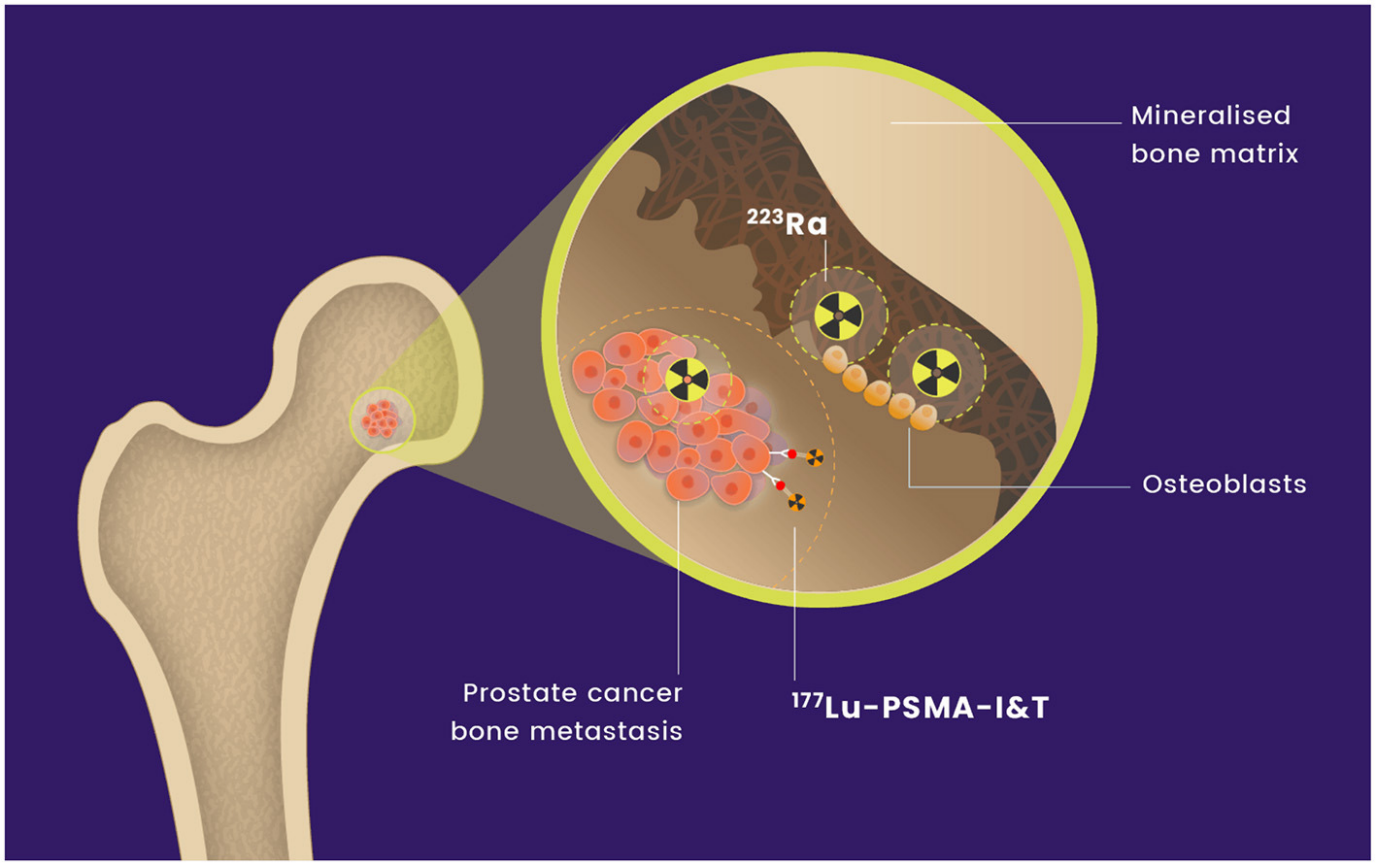
Mechanism of action of [^177^Lu]Lu-PSMA-I&T and ^223^Ra on osseous metastases.

[^177^Lu]Lu-PSMA-I&T is currently being evaluated in combination with ^255^Ac-J591, a PSMA-directed monoclonal antibody radiolabelled with an alpha-emitter (NCT04886986). [^177^Lu]Lu-PSMA-I&T has not previously been combined with ^223^Ra. We hypothesize that the combination of [^177^Lu]Lu-PSMA-I&T and ^223^Ra will deliver effective radiation to sites of metastatic prostate cancer with an acceptable safety profile (see Figure 1). We anticipate that this combination will be synergistic and lead to higher and more durable responses through more effective treatment of micrometastatic marrow disease.

The physiologic bio-distribution of ^223^Ra and [^177^Lu]Lu-PSMA-I&T is non-overlapping, further supporting our rationale for combining these radionuclides. ^223^Ra and [^177^Lu]Lu-PSMA-I&T have different methods of clearance (fecal and renal, respectively). Bowel uptake by both tracers is a potential overlapping toxicity, although the binding sites are different with specific small bowel uptake with [^177^Lu]Lu-PSMA compared to fecal excretion with ^223^Ra (22). It is possible, however, that overlapping toxicities will occur with this combination, with myeloid toxicity being of greatest concern. For [^177^Lu]Lu-PSMA, the incidence of grade 3 or higher anemia, neutropaenia and thrombocytopaenia is in the range of 8–13, 2.5–7, and 8–13%, respectively, based on pooled data from the LuPSMA (6), TheraP (2) and VISION (3) trials. Given this, the frequency of anemia and thrombocytopaenia in particular may be higher when combined with ^223^Ra. Due to this, a traditional 3+3 dose escalation model will be utilized initially, as described below.

### AlphaBet study design

**Figure 2:**
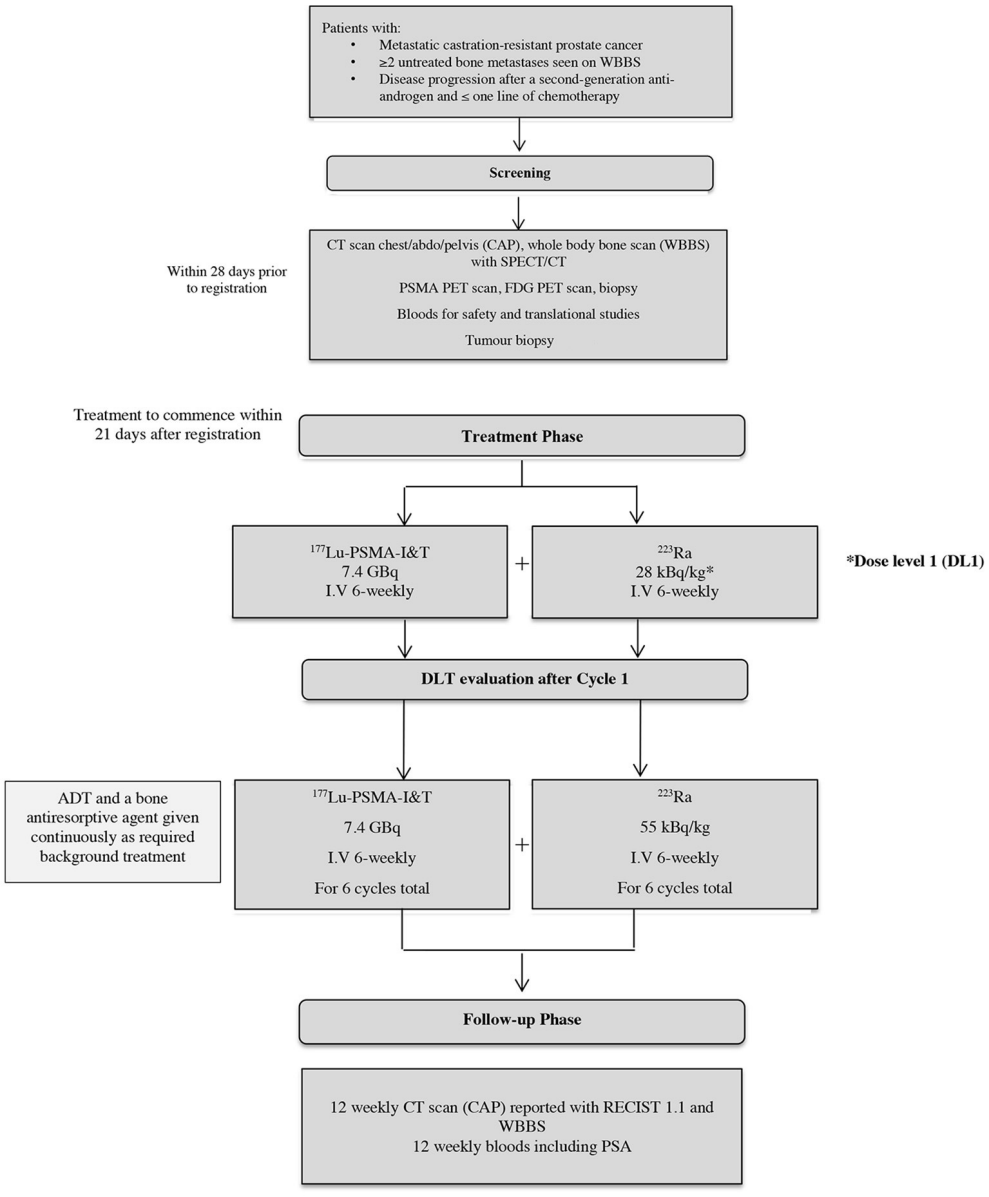
Study schema.

The AlphaBet study is a single-center, single-arm, phase I/II clinical trial evaluating the combination of ^223^Ra with [^177^Lu]Lu-PSMA-I&T in men with mCRPC who have progressed on a prior ARI. We aim to recruit approximately thirty-six patients over the course of 24 months. The chosen sample size was pragmatic, and sufficient to determine the maximum tolerated dose (MTD). The dose of [^17^^7^Lu]Lu-PSMA-I&T will be fixed at 7.4 GBq every six weeks, whereas the dose of ^223^Ra will be escalated in a two-step process in the first phase of this trial (range 28 kBq/kg−55 kBq/kg every six weeks). The study schema is demonstrated in Figure 2.

This investigator-initiated study is sponsored by the Peter MacCallum Cancer Centre (PMCC), and ethics approval has been obtained from the PMCC Human Research Ethics Committee (HREC) in July 2022. This study was financially supported by Bayer and the Peter MacCallum Cancer Foundation, in addition to a Prostate Cancer Foundation (PCF) grant. The funders had no input into the trial design. The trial is registered at clinicaltrials.gov (NCT05383079).

The co-primary aims of the study are to determine the MTD and RP2D of ^223^Ra when combined with [^177^Lu]Lu-PSMA-I&T, as well as the 50% PSA response rate (PSA-RR) for all patients treated at the MTD. See Table 2 for secondary and exploratory objectives.

**Table 2:** Secondary and exploratory objectives.

**Secondary objectives**	• To evaluate the safety of ^223^Ra in combination with [^177^Lu]Lu-PSMA-I&T in patients with mCRPC through assessing the frequency and severity of AEs as per Common Terminology Criteria for Adverse Events version 5.0 (CTCAE v 5.0). • Radiographic PFS (rPFS). • PSA-PFS. • PFS. • OS. • Objective Response Rate (ORR). • To evaluate changes in health-related quality of life (HR-QoL) using FACT-P and pain using BPI-SF within 12 months of treatment commencement.
**Exploratory objectives**	• Time to ALP response. • Time to ALP progression. • Associations between imaging (PSMA PET/CT, FDG PET/CT, bone scan SPECT/CT, and post therapy SPECT-CT) and baseline characteristics and outcomes. • Dynamic changes in circulating tumor DNA (ctDNA) fraction and utility of ctDNA genomic aberrations as a predictive biomarker of response. • Changes to circulating and tumor infiltrating immune cells post therapy and their association with clinical outcome.

### Study population

Patients eligible for this study have mCRPC which has progressed after prior treatment with an ARI. Patients must have at least two untreated bone metastases visible on bone scintigraphy, PSMA-avid disease (SUVmax ≥20), and no discordant disease on FDG PET imaging (unless discordant lesions have increased uptake on bone scintigraphy). The full inclusion and exclusion criteria are detailed in Table 3.

**Table 3:** Inclusion and exclusion criteria.

**Inclusion criteria**	1. Patient has provided written informed consent. 2. Male patients must be 18 years of age or older at the time of written informed consent. 3. Histologically or cytologically confirmed adenocarcinoma of the prostate OR unequivocal diagnosis of metastatic prostate cancer (i.e., involving bone or pelvic lymph nodes or para-aortic lymph nodes) with an elevated serum prostate specific antigen (PSA). 4. Eastern Cooperative Oncology Group (ECOG) performance status of ≤ 2. 5. Patients must have progressed on a ≥1 second-generation androgen receptor (AR)-targeted agent (e.g., enzalutamide, abiraterone, darolutamide, or apalutamide). 6. Patients must have progressive disease for study entry defined as any one of the following: • PSA progression: minimum of two rising PSA values from a baseline measurement with an interval of ≥ 1 week between each measurement. • Soft tissue progression as per Response Evaluation Criteria in Solid Tumors version 1.1 (RECIST 1.1) criteria. • Bone progression: ≥ 2 new lesions on bone scan. • Symptomatic progression e.g., bone pain. 7. At least 3 weeks since the completion of systemic therapy, surgery, or radiotherapy prior to registration. 8. Prior surgical orchiectomy or chemical castration maintained on luteinizing hormone-releasing hormone (LHRH) analog (agonist or antagonist). 9. Serum testosterone levels ≤ 1.75nmol/L within 28 days prior to registration. 10. Significant PSMA avidity on PSMA PET/CT, defined as a minimum uptake of maximum standardized uptake value (SUVmax) of 20 at a site of disease, and SUVmax ≥10 at sites of measurable disease ≥10 mm (unless subject to factors explaining a lower uptake, e.g., respiratory motion, reconstruction artifact). 11. The presence of ≥ 2 bone metastases on bone scintigraphy, which have not been previously treated with radiotherapy. 12. No contraindication to treatment with a bone antiresorptive agent such as denosumab or zoledronic acid. 13. Patients must have adequate bone marrow, hepatic and renal function documented within 28 days prior to registration, defined as: • Hemoglobin ≥ 90 g/L independent of transfusions (no red blood cell transfusion in last four weeks) • Absolute neutrophil count (ANC) ≥ 1.5 x 10^9^/L • Platelets ≥ 150 x 10^9^/L • Total bilirubin ≤ 1.5 x upper limit of normal (ULN) except for patients with known Gilbert's syndrome, where this applies for the unconjugated bilirubin component • Aspartate transaminase (AST) and alanine transaminase (ALT) ≤ 2.5 x ULN if there is no evidence of liver metastasis or ≤ 5 x ULN in the presence of liver metastases • Albumin ≥ 25 g/L • Adequate renal function: patients must have a creatinine clearance estimated of ≥ 40 mL/min using the Cockcroft Gault equation. 14. Sexually active patients are willing to use medically acceptable forms of barrier contraception. 15. Willing to undergo biopsies if disease is considered accessible and biopsy is feasible. 16. Willing and able to comply with all study requirements, including all treatments and the timing and nature of all required assessments.
**Exclusion criteria**	1. Superscan on whole body bone scan (WBBS) or diffuse marrow disease on PSMA PET. 2. Prior treatment with ^223^Ra or [^177^Lu]Lu-PSMA. 3. Has received more than one previous line of chemotherapy for the treatment of metastatic prostate cancer. 4. Site(s) of discordant FDG positive disease defined by minimal PSMA expression and no uptake on whole body bone scan ([WBBS] for bone metastases). 5. Other malignancies (in addition to the prostate cancer being treated in this study) within the previous 2-years prior to registration other than basal cell or squamous cell carcinomas of skin or other cancers that are unlikely to recur within 24 months. 6. Symptomatic brain metastases or leptomeningeal metastases. 7. Patients with symptomatic or impending cord compression unless appropriately treated beforehand and clinically stable for ≥ 4 weeks. 8. Concurrent illness, including severe infection that may jeopardize the ability of the patient to undergo the procedures outlined in this protocol with reasonable safety.

### Treatment

In the dose-escalation phase of this study, patients will receive 7.4 GBq of [^177^Lu]Lu-PSMA-I&T in combination with escalating doses of ^223^Ra, both given intravenously every six weeks. The [^177^Lu]Lu-PSMA-I&T will be given on day 1 of a six-week cycle, and ^223^Ra administered after the [^177^Lu]Lu-PSMA-I&T between days 1–5. A maximum of six cycles will be administered in total, in line with previous clinical trials evaluating [^177^Lu]Lu-PSMA (2, 3). The total number of cycles administered for each patient will be determined by the treating investigators, and take into account PSA response, post-treatment SPECT/CT imaging, and any toxicities experienced. Treatment may be paused early in the setting of an exceptional response (see below–Treatment Discontinuation). All patients will receive concomitant bone protective therapy whilst on this trial, either with denosumab or zoledronic acid, in addition to ongoing androgen deprivation therapy (ADT). Patients will receive ondansetron (or equivalent) on days 1–3 of each cycle and additional antiemetics as required.

Dose escalation will employ a traditional 3 + 3 design to assess the safety and MTD of ^223^Ra in combination with [^177^Lu]Lu-PSMA-I&T. There are 2 planned dose levels of ^223^Ra (Table 4) that will be evaluated in conjunction with 7.4 GBq of [^177^Lu]Lu-PSMA-I&T.

**Table 4:** ^223^Ra planned dose levels.

**Dose level**	**^223^Ra**
Dose level 1	28 kBq/kg
Dose level 2	55 kBq/kg

In the dose expansion phase, up to 27 patients will be treated at the determined MTD or maximum administered dose (MAD), to provide further characterization of the safety and efficacy of ^223^Ra and [^177^Lu]Lu-PSMA-I&T in combination. It is possible with this treatment combination that delayed or cumulative myeloid toxicity may occur. The RP2D will be defined by all available safety data and may be less than the MTD or MAD depending on the type and severity of AEs that occur during and after the first cycle.

### Dose limiting toxicities

Hematological recovery following administration of ^223^Ra is expected within 21–28 days, and the nadir following [^177^Lu]Lu-PSMA is within 30 days. Therefore, we expect that any hematological toxicities will be resolved or improving by the end of the six-week cycle. This provides our justification for the DLT assessment period being the first six-weeks (or first cycle) of treatment.

Any of the following AEs will be considered a DLT if it occurs within 6 weeks of Cycle 1 Day 1 and is considered related to [^177^Lu]Lu-PSMA-I&T and/or ^223^Ra:

- Grade 4 neutropaenia lasting > 7 days.
  ° Granulocyte colony-stimulating factor (G-CSF) is permitted only for use in the management of febrile neutropaenia in this study.
- Grade 4 febrile neutropaenia of any duration.
- Grade ≥3 anemia lasting > 7 days, or necessitating administration of a blood transfusion for a Hb <70g/L or symptoms directly related to anemia.
- Grade 4 thrombocytopaenia lasting > 7 days, or necessitating administration of a platelet transfusion.
- Any grade ≥ 3 non-hematological AE with the following exceptions:
  ° Grade 3 tumor flare (local pain) that resolves to ≤ Grade 2 in ≤ 7 days.
  ° Grade 3 nausea, vomiting, or diarrhea that is optimally treated and resolves to Grade ≤ 2 in ≤ 5 days.
  ° Grade 3 fatigue.
- Any grade 3 or higher hematological AE resulting in an inability to deliver the second cycle of treatment.

Treatment with [^177^Lu]Lu-PSMA-I&T should be withheld during treatment-related Grade 3 or higher AEs (with the exception of fatigue or lymphocytopaenia) and not restarted until the AE has resolved to Grade 0–2 or baseline. ^223^Ra is to be delayed in conjunction with [^177^Lu]Lu-PSMA-I&T, otherwise the dose is to be omitted if required due to attributable toxicity. Dose reductions to either [^177^Lu]Lu-PSMA-I&T (20% reduction) or ^223^Ra (20–25% reduction) will be considered for treatment-related AEs of grade 3 or higher, with the exception of grade 2 xerostomia and dry eyes also warranting a dose reduction to [^177^Lu]Lu-PSMA-I&T. Up to two dose reductions of ^223^Ra and [^177^Lu]Lu-PSMA-I&T respectively are allowed. No dose re-escalations for either drug is allowed in this trial. If [^177^Lu]Lu-PSMA-I&T is discontinued due to toxicity, patients can proceed with treatment with ^223^Ra alone.

### Study assessments

#### Dosimetry

For all cycles, a post-treatment SPECT/CT will occur on Day 2, approximately 24 h after administration of [^177^Lu]Lu-PSMA-I&T. Additional SPECT/CT imaging may occur at 4, 48 and 96 hours at the discretion of the study investigators. The purpose of post-treatment SPECT/CT imaging is to estimate tumor radiation doses.

#### Patient reported outcomes

Patient Reported Outcomes (PROs) will be completed immediately prior to Cycle 1 Day 1, at six and twelve weeks, and then 12-weekly thereafter up to 48 weeks. For this study, the Functional Assessment of Cancer Therapy for Prostate Cancer (FACT-P) questionnaire will be used to describe health-related QoL, and the Brief Pain Inventory–Short Form (BPI-SF) will be used to assess pain.

#### Imaging

Patients will undergo a baseline CT chest/abdomen/pelvis and WBBS with SPECT/CT, and have both scans repeated every 12 weeks until radiographic progression, a new anti-cancer treatment is commenced, or death. After each imaging timepoint, a response assessment will be performed. RECIST1.1 will be used to assess soft tissue lesions seen on CT, and Prostate Cancer Working Group 3 (PCWG3) criteria will be used to evaluate bone lesions visible on bone scintigraphy. A repeat PSMA PET and FDG PET scan will be performed prior to Cycle 3 Day 1 for exploratory analyses only.

#### PSA

PSA will be measured every 3 weeks during treatment, and every 6 weeks from the day 21 safety visit for 48 weeks. PSA response and progression are defined according to PCWG3 recommendations (23).

#### Translational blood samples

Blood samples will be taken at baseline, prior to Cycle 2, Cycle 4 and on progression for the purposes of genomic analysis.

#### Biopsies

For patients considered to have a lesion that is safe to biopsy, a radiologically guided biopsy will occur at baseline, after 2–4 weeks from Cycle 1 Day 1, and again on progression. Biopsies will ideally be taken from the same site each time. These will be matched with serum samples taken at the same timepoints and will be used to analyse the immune response to radiotherapy.

### Follow up

After completion or discontinuation of study treatment, a 21-day safety visit will be performed. Patients will then enter the follow-up phase and continue clinical reviews and blood tests every 6 weeks for 48 weeks, at which point the reviews will then change to 12-weekly. Clinical reviews will continue until unequivocal disease progression, commencement of a new anti-cancer treatment, death, or until it has been 12 months after the last patient has completed treatment (end of trial follow-up). Additionally, PSA testing will continue until the criteria for PSA progression has been met.

### Treatment discontinuation

Reasons for study treatment discontinuation include unequivocal disease progression, unacceptable toxicity, withdrawal of consent by the patient, inter-current illness preventing further treatment, the need to start a prohibited therapy, and significant protocol non-compliance. For the purposes of this study, unequivocal progression is defined as radiographic progression (based on RECIST1.1 for soft tissue lesions and PCWG3 for bone lesions) or clinical progression (symptomatic progression and/or a need to start a new anti-cancer therapy). PSA progression alone is not considered to be unequivocal progression.

Of note, patients may also suspend treatment (both [^177^Lu]Lu-PSMA-I&T and ^223^Ra) if they demonstrate an marked reduction in uptake at all sites of disease on the 24-h post-treatment SPECT/CT scan (PSMA-uptake intensity less than liver at all sites). On progression, patients can then recommence study treatment provided they have received < six cycles in total.

### Development of biomarkers that predict patient response

The translational research arm of AlphaBet proposes to develop tumor and immune biomarkers to predict improved patient survival following combination therapy with [^177^Lu]Lu-PSMA and ^223^Ra.

Through pre-clinical work using single-cell transcriptomics and *ex-vivo* profiling, Owen et al. established that proliferating prostate cancer cells in the bone display dampened tumor cell-inherent type I interferon signaling, which renders bone metastases poorly immunogenic and treatment-resistant (24). Additionally, tumor interferon status predicts intratumoural and systemic immune reactivity, as well as radiotherapy and Immune checkpoint inhibitor (ICI) responses (25–28). We aim to measure the expression of interferon biomarkers in tumor cells pre- and post- [^177^Lu]Lu-PSMA-I&T and ^223^Ra, along with markers of immunogenicity and infiltrating immune cells. This could potentially uncover new strategies through which to predict patient response and response durability. Importantly, given that interferon signals mediate DNA damage responses upon radiotherapy, such biomarkers may be readouts of the likely benefit of [^177^Lu]Lu-PSMA-I&T and ^223^Ra before treatment commences.

Using the peripheral blood samples, the baseline, on-treatment, and progression ctDNA fractions will be analyzed and correlated with baseline patient and disease characteristics and treatment outcomes. Similarly, the genomic profile of each patient, including how this evolves throughout the trial will be analyzed. Potential biomarkers to predict for both response and resistance to treatment will be interrogated.

### Analysis plan

For the dose-escalation phase, the analysis will be focused primarily on adverse events, particularly DLTs reported in the DLT observation period. From this data, the MTD or MAD will be decided. There are 2 analyses planned for the dose-expansion phase of this study: safety analysis and final analysis. The final analysis will be performed at the completion of the study, which will be 12 months after the last patient has completed treatment, assessing all endpoints including treatment efficacy.

Descriptive statistics of baseline characteristics of all patients will be summarized overall and by trial phase. Continuous variables will be described as mean, standard deviation, interquartile range, median, minimum, and maximum, and qualitative variables will be described as counts and percentages. PSA-response rate and objective response rate will be described as percentages with 95% confidence intervals using exact methods. Survival outcomes will be described using Kaplan-Meier methods.

Pain and health related-QoL will be analyzed using linear mixed models (LMM) with time (as factor) included as a fixed effect and patient included as a random effect. The area under the curve (AUC) of relevant pain and QoL domains will be calculated using appropriate linear contrast from the LMM.

## Discussion

Given progression following [^177^Lu]Lu-PSMA-I&T is linked in many cases to micro-metastatic osseous disease, the shorter path length and high LET of ^223^Ra against bone disease provides a rationale for combining it with [^177^Lu]Lu-PSMA-I&T. These qualities, which are specific to alpha-emitters, result in a higher chance of cell death due to inducing dsDNA breaks, rather than a reliance on crossfire radiation from neighboring cells to accumulate enough cytotoxic radiation.

Due to the potential for overlapping toxicities, particularly myeloid, we opted to follow a traditional 3+3 escalation model to ensure that safety could be monitored carefully. As discussed above, we plan to dose-escalate the ^223^Ra and keep the dose of [^177^Lu]Lu-PSMA-I&T fixed as per the VISION trial (3). We have pre-specified only two dose levels for ^223^Ra (28 kBq/kg and 55 kBq/kg) given that it is well-tolerated as monotherapy. Reassuringly, a phase 2 randomized study comparing the combination of ^223^Ra with docetaxel to docetaxel alone found that the safety profile of the two groups were similar (15). In fact, febrile neutropaenia occurred more frequently in the docetaxel alone group (0% in the combination vs. 15% for docetaxel monotherapy). Any potential fracture risk from ^223^Ra, which has been observed only when combined with an ARI, will be mitigated by concurrent use of a bone-antiresorptive agent.

In terms of eligibility criteria, a minimum number of two bone lesions with increased uptake on bone scintigraphy was chosen based on the inclusion criteria from prior trials evaluating ^223^Ra (10). Patients with extensive bone metastases or diffuse marrow disease, however, were excluded as these were considered to be at increased risk of myeloid toxicity. This was defined as having a “superscan” on bone scintigraphy, which is an imaging appearance that occurs due to a high ratio of bone to soft tissue tracer accumulation, thereby diminishing renal and background soft tissue uptake. Diffuse marrow disease seen on PET scan, determined by central Nuclear Medicine review, was also excluded. This study will allow patients with discordant bone lesions (PSMA-, FDG+ on PET imaging) as defined in Table 3, as long as they have increased uptake on bone scintigraphy. Outcomes from this cohort of patients specifically will be analyzed as an exploratory endpoint.

The primary endpoint of the phase II portion of this study is PSA-RR, with survival outcomes such as OS and PFS listed as secondary endpoints. We chose this primary endpoint to enable an early assessment of disease activity, with longer follow-up required to evaluate the secondary survival outcomes. Predictive markers of response are needed to assist with future patient selection for this therapy. Similar to PSMA PET SUVmean ≥10 being a predictive imaging biomarker for response to [^177^Lu]Lu-PSMA (29, 30), exploratory analyses from the ALSYMPCA study suggest that a decline in total ALP level at 12 weeks after initiation of ^223^Ra treatment correlates with improved survival (31). This finding was corroborated in the REASSURE study (32) and therefore time to ALP response and ALP progression will also be exploratory biomarkers in our study.

With this novel combination, osseous micrometastatic disease will hopefully receive robust treatment, though an obvious limitation is that soft tissue micrometastases may remain suboptimally treated. We chose to prioritize treatment of bone lesions based on the knowledge that the bone marrow is the most common site of disease progression following [^177^Lu]Lu-PSMA therapy. Combining a PSMA radioligand with alternative alpha-emitters that are not specific to bone may overcome this limitation (eg., ^225^Ac or ^212^Pb), however this is fraught with other challenges involving manufacture and mass distribution. As previously discussed, several studies are ongoing evaluating different combinations of alpha-emitters and PSMA-based radioligands (NCT04597411, NCT05219500).

In terms of the ideal radionuclide to combine with a PSMA ligand, theoretically this would involve an isotope with high LET and a half-life matched to that of the PSMA ligand, a straightforward and reliable manufacturing process and limited toxicity of daughter isotopes. Of the potential aforementioned alpha-emitters (^212^Pb, ^223^Ra, ^225^Ac, ^211^At), all have a high LET however ^212^Pb and ^211^At are the only isotopes with a half-life similar to the PSMA ligand (10.6 and 7.2 h respectively). ^225^Ac and ^211^At are restricted by a complex production process thereby limiting supply, with ^211^At in particular having complex radiochemistry. ^212^Pb and ^211^At produce the least toxic daughter isotopes. ^212^Pb can have reliable supply as production is generator-based, so potentially this will emerge as the ideal alpha-emitter to combine with a PSMA ligand. Currently there are no studies evaluating this combination to our knowledge.

In conclusion, we hope that the AlphaBet study will be a step forward in improving outcomes for patients with mCRPC and bone metastases, and potentially inform the design of subsequent later-phase randomized studies. In particular, the exploratory translational data from tissue, blood and novel imaging will lead to a deeper understanding of the reasons and predictors for treatment response and resistance and the immune response to radiotherapy.

## Ethics statement

This study involving human participants was reviewed and approved by Peter MacCallum Cancer Centre. The patients provided their written informed consent to participate in this study.

## Author contributions

LK wrote the first draft of the manuscript. JI, TY, JX, AC, KC, KO, BP, HF, LF, JB, and MH assisted with editing and revising the manuscript. AA, JB, and MH provided oversight of the protocol and edited the manuscript. All authors contributed to the article and approved the submitted version.

## Funding

This trial is made possible through a grant from the Prostate Cancer Foundation (PCF) funded by CANICA Oslo Norway, Peter MacCallum Foundation, and support from Bayer who are providing supply of ^223^Ra and financial support. ^177^Lu, no carrier added, was supplied from the Australian Nuclear Science and Technology Organization (ANSTO). LK was supported by a Vincent Fairfax Family Foundation Research Entry Scholarship from the RACP Foundation, and LK and JB are both supported by a University of Melbourne Australian Government Research Training Program Scholarship. MH and AA are supported by NHMRC Investigator Grants.

## Conflict of interest

Author LK has the following disclosures: Honoraria for speaker duties from Bayer. Author AA has the following disclosures (lifetime): Consultant—Astellas, Janssen, Novartis, Aculeus Therapeutics; Speakers Bureau—Astellas, Janssen, Novartis, Amgen, Ipsen, Bristol Myers Squibb; Merck Serono, Bayer; Honoraria—Astellas, Novartis, Sanofi, AstraZeneca, Tolmar, Telix, Merck Serono, Janssen, Bristol Myers Squibb, Ipsen, Bayer, Pfizer, Amgen, Noxopharm, Merck Sharpe Dome, Aculeus Therapeutics; Scientific Advisory Board—Astellas, Novartis, Sanofi, AstraZeneca, Tolmar, Pfizer, Telix, Merck Serono, Janssen, Bristol Myers Squibb, Ipsen, Bayer, Merck Sharpe Dome, Amgen, Noxopharm; Travel + Accommodation—Astellas, Merck Serono, Amgen, Novartis, Janssen, Tolmar, Pfizer; Research Funding—Astellas (investigator), Merck Serono (investigator), Astra Zeneca (investigator), Bristol Myers Squibb (institutional), Astra Zeneca (institutional), Aptevo Therapeutics (institutional), Glaxo Smith Kline (institutional), Pfizer (institutional), MedImmune (institutional), Astellas (institutional), SYNthorx (institutional), Bionomics (institutional), Sanofi Aventis (institutional), Novartis (institutional), Ipsen (institutional), Exelixis (institutional), Merck Sharpe Dome (institutional), Janssen (institutional), Eli Lilly (institutional), Gilead Sciences (institutional), Merck Serono (institutional), Hinova (institutional). MH acknowledged philanthropic/government grant support from the Prostate Cancer Foundation (PCF) funded by CANICA Oslo Norway, Peter MacCallum Foundation, Medical Research Future Fund, NHMRC Investigator Grant, Movember, U.S. Department of Defense and the Prostate Cancer Foundation of Australia (PCFA). Author MH acknowledges grant support from AAA/Novartis, ANSTO, Bayer, Isotopia. Consulting fees for lectures or advisory boards from Astellas, AstraZeneca, Janssen, Merck/MSD, Mundipharma and Point Biopharma. The remaining authors declare that the research was conducted in the absence of any commercial or financial relationships that could be construed as a potential conflict of interest. The authors declare that this study received funding from Bayer. The funder was not involved in the study design, the writing of this article or the decision to submit it for publication.

## Publisher's note

All claims expressed in this article are solely those of the authors and do not necessarily represent those of their affiliated organizations, or those of the publisher, the editors and the reviewers. Any product that may be evaluated in this article, or claim that may be made by its manufacturer, is not guaranteed or endorsed by the publisher.
